# Roles of Histone Deacetylases and Inhibitors in Anticancer Therapy

**DOI:** 10.3390/cancers12061664

**Published:** 2020-06-23

**Authors:** Flávia Alves Verza, Umashankar Das, Ana Lúcia Fachin, Jonathan R. Dimmock, Mozart Marins

**Affiliations:** 1 Biotechnology Unit, University of Ribeirão Preto, Ribeirão Preto SP CEP 14096-900, Brazil; flaviaverza@hotmail.com (F.A.V.); afachin@unaerp.br (A.L.F.); 2 College of Pharmacy and Nutrition, University of Saskatchewan, 110 Science Place, Saskatoon, SK S7N 5C9, Canada; umashankar.usask@gmail.com; 3 Medicine School, University of Ribeirão Preto, Ribeirão Preto SP CEP 14096-900, Brazil; 4 Pharmaceutical Sciences School, University of Ribeirão Preto, Ribeirão Preto SP CEP 14096-900, Brazil

**Keywords:** histone deacetylases, histone deacetylase inhibitors, histones, cancer, curcumin, chalcones, histone acetyltransferase

## Abstract

Histones are the main structural proteins of eukaryotic chromatin. Histone acetylation/ deacetylation are the epigenetic mechanisms of the regulation of gene expression and are catalyzed by histone acetyltransferases (HAT) and histone deacetylases (HDAC). These epigenetic alterations of DNA structure influence the action of transcription factors which can induce or repress gene transcription. The HATs catalyze acetylation and the events related to gene transcription and are also responsible for transporting newly synthesized histones from the cytoplasm to the nucleus. The activity of HDACs is mainly involved in silencing gene expression and according to their specialized functions are divided into classes I, II, III and IV. The disturbance of the expression and mutations of HDAC genes causes the aberrant transcription of key genes regulating important cancer pathways such as cell proliferation, cell-cycle regulation and apoptosis. In view of their role in cancer pathways, HDACs are considered promising therapeutic targets and the development of HDAC inhibitors is a hot topic in the search for new anticancer drugs. The present review will focus on HDACs I, II and IV, the best known inhibitors and potential alternative inhibitors derived from natural and synthetic products which can be used to influence HDAC activity and the development of new cancer therapies.

## 1. Introduction

**Figure 1:**
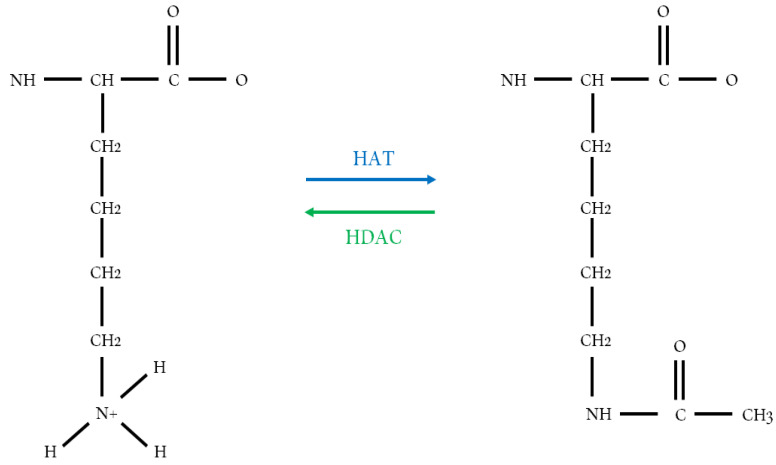
Modifications in the structure of histones mediated by histone acetyltransferase (HAT) and histone deacetylase (HDAC). The figure shows the lysine residue undergoing acetylation (HAT) and deacetylation (HDAC).

The mechanisms regulating gene expression involve a series of molecular modifications in DNA and chromatin and are responsible for the response to any type of physiological signaling in the organism [1]. This constant maintenance requires specific levels of control, and is undertaken by transcriptional regulators which bind to selected sequences in the DNA, inducing the production of proteins responsible for the structural modifications in chromatin and the consequent binding of regulators to DNA, causing epigenetic modifications [2]. Chromatin consists of the association of double-stranded DNA and proteins whose basic unit is the nucleosome. Chromatin can be divided into euchromatin, which corresponds to transcriptionally active DNA, and heterochromatin, which consists of inactive DNA and appears to exert structural functions during the cell cycle, allowing the cell to have control of the genes present in its nucleus [3,4]. The nucleosome core consists of 146 bp of DNA wrapped around a histone octamer. Histones are the main structural proteins associated with DNA in eukaryotic cells [5]. These proteins are divided into two groups: core histones, including H2A, H2B, H3 and H4, and linker histones (H1/H5) [6]. The four core histones H2A, H2B, H3 and H4 are assembled into H2A/H2B dimers and H3/H4 tetramers, forming the octamer complex composed of two H2A/H2B dimers and one H3/H4 tetramer, while the linker histone H1 binds to the DNA entry or exit sites on the surface of the nucleosomal core particle and completes the nucleosome [6,7]. These nuclear histones are small basic proteins composed of a large number of amino acids, mostly lysines and arginines [8]. Histones possess side chains that can be the target of covalent modifications such as acetylation, the mono-, di- and tri-methylation of lysins and the phosphorylation of serines [9]. Several of these side chain modifications can occur in the N-terminal tail domains of histones located outside the nucleosome, thereby weakening the binding between histones and DNA [10] and between regulatory proteins and histones [11,12]. This weakened binding can facilitate the action of transcription factors in this altered region, inducing or repressing gene transcription [13]. Specific modifications can also occur in the side chains of the internal histones of the nucleosome [14]. Histone modifications catalyzed by enzymes use the energy of ATP hydrolysis to modify nucleosomes [15] and this recruitment of complexes with specific enzymatic activities can influence gene transcription, replication, repair and recombination [14]. The tail domains of core histones can be modified by acetylation, phosphorylation, methylation, ubiquitination and sumoylation and less frequently, by citrullination and ADP-ribosylation [16]. These post-translational modifications alter the interactions between DNA and histones or the binding of proteins and transcription factors to chromatin [17]. Histone acetylation is a post-translational modification with functional implications for different cellular processes [18]. The presence of acetylated lysine in the histone tail results in a relaxed chromatin state, allowing the activation of gene transcription in that region. On the other hand, the deacetylation of lysine residues is associated with condensed chromatin, which impedes the transcription of genes present in that chromatin region [19,20]. The acetylation and deacetylation of lysine residues is controlled by two enzymes with opposite activities involved in gene regulation [21]. These reactions are catalyzed by enzymes with “histone acetyltransferase” (HAT) or “histone deacetylase” (HDAC) activity, which add or remove acetyl groups, respectively [22] (see Figure 1).

Different forms of HAT and HDAC have been identified, including coactivators that can interact with transcription regulators [23]. Deacetylation increases the ionic interactions between positively charged histones and negatively charged DNA, which produces a more compact chromatin structure, repressing gene transcription by preventing the access of the transcription machinery to the site. On the other hand, histone acetylation has been associated with other functions of the genome, such as producing a looser chromatin assembly that influences DNA repair and recombination [24,25]. The modifications in the histone chains define the compaction or relaxation of chromatin through the recruitment and binding of various specific proteins, functioning as an epigenetic machinery [26]. This epigenetic information is an important component in the regulation of gene expression since the breakdown of epigenetic integrity has been associated with different diseases, including cancer [27,28]. The addition of an acetyl group to a lysine residue neutralizes its positive charge on the nitrogen atom, alters the structure of the amino acid and blocks other modifications at this specific site [29]. This epigenetic alteration is related to disorders and diseases because of its participation in various physiological processes, acting in synergy with transcription factors, oncoproteins and kinases, affecting protein stabilization and activating or inhibiting gene transcription and DNA repair [30]. 

The HATs are divided into two classes (type A and type B) according to their localization and function in the cell. Their activity can be modulated by several protein–protein interactions, protein cofactors and autoacetylation. Type A HATs are nuclear enzymes which contain acetyl-CoA binding sites. This class of HATs probably catalyzes acetylation and events related to gene transcription. Type B HATs are believed to have a maintenance role in cell functioning as cytoplasmic enzymes that modify free histones in the cytoplasm after their synthesis and transport them to the nucleus, where they can be deacetylated and incorporated into chromatin [31]. In addition, HATs can be divided into four families based on their primary structure: GNAT (Gcn5, PCAF, Hat1, Elp3 and Hpa2); p300/CBP (p300 and CBP); MYST (Esa1, MOF, Sas2, Sas3, MORF, Tip60 and Hbo1) and Rtt109 [31,32]. One specific HAT can acetylate several lysine residues of a histone. Many HATs contain bromodomains for the recognition of the acetylated lysine. The acetyl lysine residues in the histone tails form bromodomain binding sites where the adjacent amino acids determine specificity. This indicates that acetylation, like many protein phosphorylation events, and creates a new binding surface to recruit other proteins to the nucleosome [33]. Histone acetylation occurs throughout the cell cycle and differs from histone methylation, which has greater activity in the G2 phase [34]. The acetylation pattern of certain lysine residues in the histone tails appears to result from the opposite activities of HATs and HDACs. These changes in the acetyl group can be quickly reversed in some chromatin environments, suggesting that the ‘transient’ nature of gene expression may be linked to the degree of acetylation. Even though acetylation and histone methylation are dynamic and involved in several biological processes, little is known about how the different functional domains of chromatin are established and maintained [35]. Finally, the activity and specificity of these acetyltransferases can be altered by factors such as autoacetylation proteins and chaperones [36,37]. 

**Figure 2:**
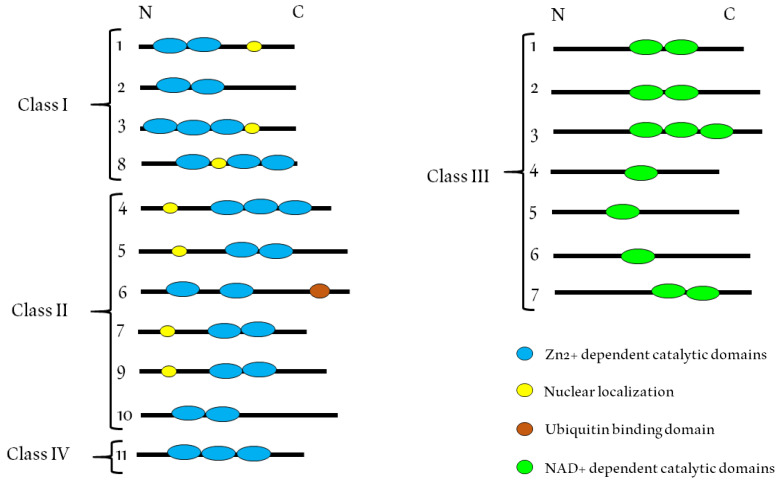
Schematic organization of the HDAC classes I, II and IV showing the Zn^2+^-dependent catalytic domains in blue, the NAD^+^-dependent catalytic domains in pink, the nuclear localization in yellow and the ubiquitin-binding domain of HDAC6 in orange.

HDACs are a family of enzymes that play important roles in different biological processes, mainly because of their gene transcription-repressing activity [38]. These enzymes are able to remove acetyl groups (O=C-CH_3_) from an ε-N-acetyl lysine in a histone, an event that confers to histones the capacity to compact DNA [39]. The enzymatic activity that catalyzes the deacetylation of histones was discovered for the first time in 1969 [40]. HDACs possess a catalytic domain that requires a Zn^2+^ ion (classes I, II and IV) [41] or NAD+ (class III) [41] (see Figure 2).

Eighteen HDACs have been identified in humans (Table 1), which are divided into four classes: class I Rpd3-like proteins (HDAC1, HDAC2, HDAC3, and HDAC8) which have a nuclear localization. Class IIa (HDAC4, HDAC5, HDAC7, and HDAC9) and IIb (HDAC6 and HDAC10) Hda1-like proteins which show a specific expression in tissue and can be transported between the nucleus and cytoplasm, suggesting the involvement of this HDAC class in the acetylation of non-histone proteins [42]. Class III are Sir2-type proteins (SIRT1, SIRT2, SIRT3, SIRT4, SIRT5, SIRT6, and SIRT7). The specific expression pattern of this class is unknown, and its mechanisms differ from those of the other two classes and are not in the scope of this review. The protein of class IV (HDAC11) shows homology to both class I and class II members [43]. Table 1 summarizes these three related classes. Evidence suggests that HDACs can also deacetylate non-histone proteins such as hormone receptors, chaperones and cytoskeletal proteins that regulate cell proliferation and death [43,44,45,46]. The HDACs can form gene silencing complexes with nuclear receptors when a specific ligand is absent [47]. Studies have indicated that HDACs can regulate the expression of various genes through the interaction with transcription factors such as E2f, Stat3, p53, NF-κB and TFIIE [48]. The absence of HDAC1 leads to reduced deacetylase activity and the hyperacetylation of other histones. In addition, an increase in HDAC2 and HDAC3 expression is observed in HDAC1-deficient cells, which is unable to compensate for the loss of HDAC1, suggesting a unique function of this enzyme [49]. HDAC4 has been described as an important regulator of chondrocyte hypertrophy during skeletogenesis and a general role of class II HDACs in the control of cellular hypertrophy has also been suggested [50,51].

HDAC1 and HDAC2 were discovered in 1996 [69,70], while HDAC3 was described in 1997 [71]. HDAC4, 5 and 6 were characterized in the same year, HDAC7 in 1999 [72] and HDAC8 in 2000 [73,74]. Many different forms of HDACs can arise from nucleotide polymorphisms or alternative splicing. For example, different isoforms of HDAC9 have been described [75]. Although HDAC4, HDAC5, HDAC7 and HDAC9 have similar functions in the regulation of cytoplasmic-nuclear transport and DNA binding, they are encoded by different genes and are not isoforms [41,76]. HDAC10, on the other hand, has no association with other proteins forming complexes, which indicates that the involvement in the transcription control occurs by other means [77]. Like HDAC10, HDAC11 was discovered in 2002 [78] and so far they are the least studied and understood HDACs.

## 2. Class I HDACs

Class I HDACs are multiprotein complexes (except for HDAC8) that can be expressed simultaneously at different sites [39]. Enzymes of this class are involved in cell proliferation and survival [38,79]. HDACs 1 and 2 are found in the complex that represses the expression of neuronal genes in non-neuronal tissues [80]. HDAC1 was found to exert a protective function against the formation of teratomas with malignant potential in mice and human patients [81]. HDAC2 negatively regulates memory formation and synaptic plasticity [82]. HDAC1 and HDAC2 repress the expression of proteins p21 and p57 which regulate the transition from the G1 to the S phase of the cell cycle in fibroblasts [83]. HDAC3 also has a repressive function when it interacts with other molecules forming an enzyme complex, in addition to playing an important role among class I HDACs in gene expression in inflammation [84]. The activity of HDAC8 was found to be dependent on oxidoreduction reactions [85].

### 2.1. The HDAC1/2 Functional Complex

HDAC1 and HDAC2 are homologous proteins (82–85% similar in human proteins) and are part of stable multiprotein complexes [86,87]. These complexes account for about 50% of all deacetylase activity in embryonic stem cells [88] and T cells [89]. HDAC1/2 cannot bind directly to DNA without interacting with other specific molecules and its activity was reduced in the absence of this binding [90]. These molecules can be transcription regulators, transcription factors and DNA-binding factors, as well as coactivator and corepressor complexes that alter the chromatin structure [91]. Corepressors act on transcriptional silencing by recruiting promoters of chromatin remodeling that can inhibit or silence the basal transcription mechanism [92]. Some of the known HDAC1/2 corepressor complexes are Sin3A, nucleosome remodeling and deacetylase (NuRD), CoREST, mitotic deacetylase (MiDAC) and SMRT/NCor, which are recruited to chromatin by transcription factors [2,9,93,94,95]. As described below, the structures of Sin3A and NuRD exemplify the diversity of sizes and the number of subunits of these complexes. This diversity and the incorporation of sequence-specific DNA-binding proteins can modulate their function and cellular context activity [96]. It is extremely important that the machinery involved in the deacetylation of histones is not only mechanically regulated but also with high specificity. Even today, little is known about how this specificity is achieved; however, studies have indicated that different multiprotein complexes are involved in this regulation.

### 2.2. Sin3A Complex

**Figure 3:**
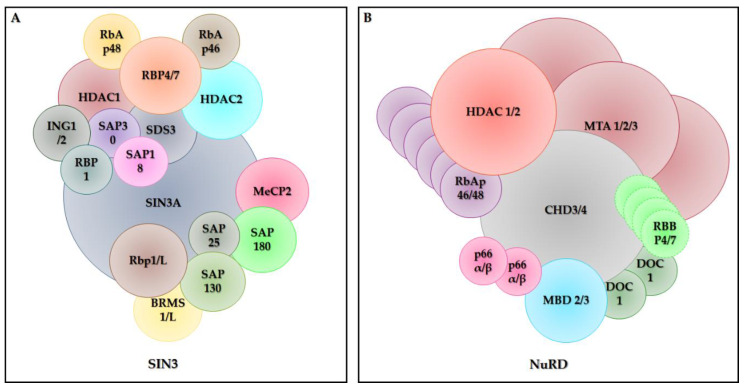
Structure of class I HDAC co-repressor Complexes. HDAC1 and HDAC2 are recruited to the (**A**) SIN3 and the (**B**) NuRD. Adapted from [96,100,115].

The Sin3/HDAC corepressor complex is a multiprotein complex that mediates gene repression by recruiting HDACs (class I, especially HDAC1 and HDAC2) and other chromatin-modifying enzymes [97] and also acts as a coactivator and general transcription factor [98]. The Sin3A core complex includes HDAC1, HDAC2, Sin3a, RbAp46 and RbAp48, RbAp4, RbAp7, SAP30, SAP18 and SDS3 [96,99,100]. Besides these core proteins, several other proteins have been associated to this core complex and include SAP180 [101], RBP1 [102], BRMS1 [103], SAP130 [101], SAP25 [104], MeCP2 [105] and ING1/2 [100] (see Figure 3A). These factors are essential for the Sin3A complex and exert their function through different types of interaction mediated by amphipathic helical domains that contain a polar and an apolar end and conserved segments [106]. Although structurally similar, the amphipathic helical domains and conserved segments have binding specificity [107]. The Sin3A complex has also been associated with other enzymatic activities depending on the molecules of the interaction [87,108,109]. This complex plays an important role in maintaining the pluripotency of embryonic stem cells [110]. On the other hand, the acetylation of STAT3 and its association with Sin3A contribute to the oncogenic potential of STAT3 [111]. The absence of the Sin3 complex can result in positive and negative gene regulation [112,113]; however, its interaction mode with these regulators still remains unknown.

### 2.3. NuRD Complex

The nucleosome remodeling deacetylase complex (NuRD), also known as Mi-2, according to stoichiometry data [114,115], is composed of one copy of the CHD3 (Mi2α) or CHD4 (Mi2β) proteins (chromodomain, helicase, DNA binding domain), one HDAC1 or HDAC2, three MTA1/2/3 (metastasis associated), one copy of MBD2 and MDB3 proteins (Methylated CpGBinding), six copies of RbAp46/48 proteins (retinoblastoma associated protein), two GATAD2a/b (p66a/b) and two DOC-1 (deleted in oral cancer) [115]. Other studies have also indicated that four molecules of RBBP4/7 (4/7 retinoblastoma binding protein) integrate the NuRD core complex [116,117] (see Figure 3B). This protein complex is conserved in animals and is widely expressed in most tissues, influencing gene transcription, chromatin assembly, cell cycle progression and genomic stability [118]. The role of the NuRD complex is determined by the combination of the six main protein subunits that make up this complex [119]. Several functional differences exist between the enzymes that form the NuRD complex, a fact that interferes with the specialized functions of the complex, which can act in different types of cells and biological systems. For example, MBD2 and MBD3 are related proteins with a methyl-CpG consensus binding domains that are found exclusively in NuRD complexes [120]. MBD2 recognizes and binds to methylated DNA, while MBD3 contains an amino acid alteration that prevents this binding [121,122]. In addition to their functions within the NuRD complex, some subunits of this complex such as MBD3 can serve as a protein interaction domain and bind to other protein complexes, for example the JUN oncoprotein [123]. The NuRD complex also regulates how DNA is read in different cells. This feature is extremely important to transform adult cells into induced pluripotent stem cells (iPSCs), an epigenetic change that may be used to treat different diseases [124]. 

These complexes show a diversity in the sizes and numbers of subunits. Larger complex formation is required for better activity. The MiDAC and CoREST complexes also contain HDAC I and II enzymes. MiDAC is a tetrameric complex that is composed of HDAC1/2, DNTTIP1 (deoxynucleotidyltransferase terminally interacting protein 1) and the protein co-repressor MIDEAS (SANT domain associated with mitotic deacetylase) [125,126]. The CoREST complex contains a single copy of CoREST1/2/3, LSD1 demethylase (specific lysine demethylase 1) and HDAC1/2 proteins [127]. NCoR/SMRT is associated with HDAC3 and contains transducin β-like protein 1 (TBL1)/TBL1-related protein 1 (TBLR1) and G-protein pathway suppressor 2 (GPS2) [96]. Class II HDAC activity is dependent on the interaction with the SMRT/NCoR–HDAC3 complex [128].

## 3. Class II HDACs 

Class II HDACs (HDAC4, 5, 6, 7, 9 and 10) are found in the nucleus and cytoplasm (see Table 1) and can freely shuttle between these two compartments, exhibiting specific functions in tissues [38,79]. For example, HDACs 4, 5 and 7 regulate cell differentiation according to a specific signal, which results in changes in gene expression [39]. This class of HDACs can be subdivided into class IIa (HDAC4, 5, 7, and 9) and class IIb (HDAC6 and 10) [38]. Class II HDACs do not only act as transcription repressors but also interact with non-histone substrates, inducing autophagy and regulating the microtubules of the cytoskeleton [129].

Class IIa HDACs have low enzymatic activity but can recruit other protein complexes, exerting deacetylase function [128]. HDACs of this class can induce the conversion of cell signaling by presenting conservative serine residues in the regulatory N-terminal domains, with reversible phosphorylation [130]. This phosphorylation leads to the activation of several kinases and phosphatases which, when functioning downstream of biological pathways, regulate the transit of HDACs between the cytoplasm and nucleus, as well as their binding to DNA [131]. The phosphorylation of class IIa HDACs is crucial for the determination of their localization and transcriptional repression capacity in the nucleus. For example, in the nucleus, HDAC9 represses proteins such as myocyte enhancer factor-2 (MEF2) until a myogenic differentiation signal causes its export to the cytoplasm [130]. Class IIa HDACs have been shown to exert their transcriptional repressive function in different tissues such as skeletal, cardiac and smooth muscles, bone, immune system, vascular system and brain [132,133]. Most known HDAC inhibitors do not affect class IIa HDACs [134]. 

Class IIb HDACs (HDAC6 and HDAC10) possess duplicated catalytic domains and are usually localized in the cytoplasm [134]. HDAC10 is very similar to HDAC6, with both forms containing a second catalytic domain that is not found in other HDACs. However, in HDAC10, this domain has no known function [135]. HDAC10 together with HDAC9 has been shown to be necessary for homologous recombination activity, but it remains unclear whether they are direct participants in this process or act through transcriptional control [136]. HDAC6 catalyzes the deacetylation of α-tubulin and promotes microtubule- and actin-dependent cell motility. In addition, this enzyme plays a critical role in the clearance of misfolded proteins by inducing autophagy [137]. HDAC6 contains two catalytic sites and a ubiquitin-binding domain (see Figure 1). The latter is important for the response to cytotoxic protein aggregates [138]. HDAC6 is an important potential therapeutic target for the treatment of diseases such as Alzheimer’s and cancer [139].

Class I and II HDACs show great sequence similarity in proteins of the same class, directed to the catalytic site. For example, HDACs 1, 2 and 3 have a homologous sequence and can be expressed together, but only HDACs 1 and 2 together form other co-repressor complexes in the cell while HDAC3 joins other proteins to exert its repressive activity [140]. Except for HDAC8, all other HDAC isoforms are always found associated with other proteins, or other HDAC isoforms, in multiprotein complexes [141]. In the same cell, there may be similar isoforms being expressed that may be part of different DNA binding complexes. The homologous proteins of HDAC1, HDAC2 and HDAC3 are regulated in the absence of HDAC1, but mammalian cells need to maintain specific levels of deacetylase activities to ensure the uninterrupted and efficient cell cycle progression. We can also observe that the loss of HDAC1 results in an increase in acetylation at its specific activity site, which can affect the expression of specific genes [49]. 

Specific patterns of histone acetylation and deacetylation do not occur by chance, they are influenced by other modifications of histones. These post-translational modifications together generate a ‘histone code’ [142]. For example, the acetylation of histone H3-K9 and the methylation of H3-K4 are associated with active transcription. The loss of the acetylation of histone H3-K9 and the gain of the methylation of H3-K9 and H3-K27 are indicative of heterochromatin [143]. In many cases, the modifications of a single residue are mutated exclusively. The presence of a modification can induce additional modifications in nearby amino acids, expanding the change in the protein coding information. Gene promoters suppressed by the HDAC inhibitors generally contain hypermethylated DNA, indicating interference between the histone acetylation/methylation and DNA methylation. Each of these events can have profound implications for gene expression in normal and cancerous cells [144].

## 4. Class IV HDACs

HDAC11 is the only class IV HDAC. The expression of this enzyme was observed in some tissues such as the brain and heart, but little is known about its function [78,145]. HDAC11 is related to immune system regulation since the suppression of this deacetylase promoted the expression of IL-10 in animals [146].

HDACs have been studied extensively, especially because of their role in cancer. In contrast, no clinical applications have been described for HATs. However, the latter may have important functions in inflammatory diseases, cancer and neurological disorders since histone acetylation results in less condensed DNA and increased gene transcription [147,148]. HATs can act on different cell substrates such as histones, transcription factors, enzymes and nuclear receptors. Despite its potential, the development of HAT inhibitors proved to be challenging and a large gap remains between the biological activity of HAT inhibitors in in vivo studies and their use as therapeutic agents, since many HAT gene knockout mutants are incompatible with life in mice [149].

## 5. Participation of Transcription Factors in the Mechanism of Regulation by HDACs

**Figure 4:**
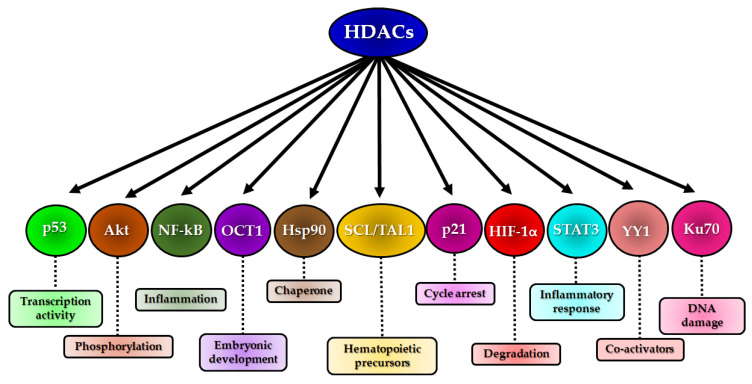
Some non-histone proteins affected by histone deacetylases (HDACs) and the changes caused by these interactions.

HDACs can exert direct effects on physiological processes such as apoptosis, differentiation, metabolism and inflammation through the deacetylation of non-histone proteins, affecting their functions, cellular localization and protein–protein interactions [150]. Some proteins such as p53, NF-κB, STAT3, Hsp90, SCL/TAL1, OCT1, YY1, Akt and Ku70 are modified by HDACs, with consequent changes in embryonic development and cell proliferation, differentiation and death [46] (see Figure 4). 

Other proteins that serve as transcription factors can also be modulated by chromatin status, limiting their capacity to bind to DNA and to activate transcription. Some transcription factors are specifically expressed in certain tissues and regulate specialized cellular functions; thus, the silencing of these transcription factors may result in a functional blockade. The deletion of the SCL/TAL1 transcription factor, for example, resulted in the inability to generate hematopoietic precursors and caused embryo death in mice [151]. Likewise, the silencing of the gene encoding transcription factor organic cation transporter 1 (OCT1) also caused disturbances in embryonic development [152]. The Yin Yang 1 (YY1) transcription factor can regulate different genes and has specific DNA-binding activity and transcriptional repression [153]; however, this transcription factor requires co-activators or co-repressors for its full function, thus interacting with HATs (CBP and p300) and class I HDACs (HDAC1, 2 and 3) [154]. Several transcription factors participate in the regulation of these pathways. These factors directly or indirectly regulate DNA repair genes which are also part of the DNA repair machinery [155]. The tumor suppressor protein p53 is an important transcription factor, particularly for the response to stress and cellular homeostasis. Under normal physiological conditions, this protein is maintained at low levels by its negative regulator MDM2 [156]. In the absence of MDM2, the levels of p53 increase, causing embryonic lethality in mice [157]. The acetylation of p53 at different lysine residues is mediated by p300/CBP, increasing its DNA-binding capacity and the consequent transcription of its target genes [158,159]. This even occurs in response to cell damage but is transitory and reversible, as the post-translational modifications maintain p53 acetylation under control [160]. 

During the response to genotoxic stress, p53 functions as a transcription factor and regulates effector genes such as GADD45A and p21 [161]. The high expression of Gadd45A can reduce the efficiency of DNA repair [162]. HDACs can decrease the activity of p53; for example, the overexpression of HDAC1 was found to reduce p53 acetylation in vivo [163]. MDM2 can recruit HDAC1, promoting the deacetylation of p53, and this silencing can more quickly interrupt the function of this protein when its target genes are no longer necessary [164].

The transcription factor Sp1 (specificity protein 1) is found in all animal cells where it directly or indirectly regulates the expression of genes such as Gadd45A and MGMT. However, in response to DNA damage, Sp1 is phosphorylated and recruited to the sites of DNA double-strand breaks where it possibly mediates the recruitment of chromatin remodeling factors involved in the repair of these breaks [165]. Sp1 can define the binding of Gadd45A at a specific site of DNA damage [166]. Sp1 is a specificity protein that belongs to the family of Krüppel transcription factors (Sp/KLF), which currently has 26 members [167]. The DNA-binding domain consists of three Cys2His2 zinc finger proteins (81 amino acids per protein), which are responsible for recognizing the GC (GGGGCGGGG) and GT/CACC (GGTGTGGGG) sequences in DNA [168]. Post-translational modifications in Sp1 modulate chromatin remodeling factors, DNA, the transcription machinery and other transcription factors to induce or repress expression [169,170]. The high expression of Sp1 has been observed in several types of cancer [170]. Sp1 can increase the activity of a gene promoter or recruit other protein complexes to exert activating or repressing functions. In breast cancer, human epidermal growth factor receptor type 2 (HER2) signaling phosphorylates Sp1, which recruits HDAC1 to the sites of gene regulation, forming a protein complex that can involve other regulators [171]. GM2 synthase is an enzyme that produces glycosphingolipids. The high expression of this enzyme is associated with a poor tumor prognosis. Its activation is regulated by histone acetylation and a reduction in the Sp1-HDAC1 repressor complex [172]. In multiple myeloma, the inhibition of HDACs was associated with the down-regulation of Sp1, indicating that the effects of HDAC expression might be mediated by Sp1 [173].

HDACs are involved in the dysregulation of pathways and transcription factors in the various phases of cancer cells. The exacerbated expression of HDACs may be one of the factors responsible for the worst prognosis for patients, with stomach and ovarian cancer [54], neuroblastoma [66] and multiple myeloma (MM) [174] for example.

Epigenetic dysregulation can nurture the onset and progression of various human diseases. HDACs induce several cellular and molecular effects through the hyperacetylation of histone and non-histone protein substrates, which are involved in the regulation of cell cycle, apoptosis, DNA-damage response, metastasis, angiogenesis, autophagy and other cellular processes being able to influence the onset or progression of diseases such as cancer [175]. However, the contribution of HDACs to this pathology may not be related to the level of expression of these proteins. HDACs can function as catalytic subunits of large protein complexes and can be recruited in ways that alter the expression of several protein genes in order to induce a tumor. The molecular and biological consequences of inhibiting HDACs need to be analyzed in this context. HDACs can be targeted using small molecules and more selective agents [176]. The inhibition of HDAC10 in combination with doxorubicin treatment decimates neuroblastoma, but not healthy cells, preventing the efflux of drugs, as well as improving DNA damage [177], and the combined genetic deletion of HDAC1 and HDAC2 results in the activation of a senescent program and the death of transformed cells [178]. HDAC1 has oncogenic activity in tumor cells, but can have different functions in different subpopulations, but the combined genetic deletion of HDAC1 and HDAC2 results in accelerated leukemogenesis. One study noted that in the pre-leukemic phase, HDAC1 blocks differentiation; compromises genomic stability; and increases self-renewal in hematopoietic progenitors, all events affected by the reduction of HDAC1 levels. The short-term treatment of pre-leukemic mice with an HDAC inhibitor (HDACi) accelerated leukemogenesis. On the other hand, the absence of HDAC1 in mice led to a longer survival time for the animals. Thus, HDAC1 has a dual role in tumorigenesis: oncosuppressive in the early stages and oncogenic in established tumor cells [179]. The authors suggest that the inhibition of HDACs may block the intrinsic antitumor functions of these proteins. However, further studies are extremely important for a greater understanding of the role of HDACs alone and together, in different stages of carcinogenesis and in different types of tumor cells. 

## 6. Inhibitors of HDACs

HDAC proteins are a promising class for drug targets due to the importance of these enzymes in a variety of processes, including cell cycle regulation, proliferation, survival, differentiation, metabolism, protein trafficking, DNA repair and angiogenesis. In recent decades, a class of inhibitors that block HDAC activity have been discovered. These inhibitors are able to inhibit gene silencing through the hyperacetylation of histones, acting on the regulation of gene expression and influencing cell growth and differentiation and the induction of apoptosis in neoplasms [180]. Numerous synthetic or natural molecules that aim at classes I, II and IV enzymes have been developed and characterized, although interest in the class III Sirtuin family is increasing. Class I, II and IV exhibit their Zn^2+^-dependent deacetylase activity. The binding of HDACi to this ion, found in the active site of HDACs, alters the deacetylase activity of these proteins and damages their enzymatic function [181]. As the expression of HDACs is not organized in several types of cancer, the disrupted balance between HATs and HDACs in neoplastic cells can contribute to carcinogenesis and the reversible modulation function of HDACs makes these proteins interesting targets for cancer treatment [41]. HDACi can neutralize the abnormal acetylation status of proteins found in cancer cells and can reactivate the expression of tumor suppressors. Cancer cells may also be more sensitive to HDACi-induced apoptosis than normal cells, enhancing the therapeutic potential of HDACi [182]. 

**Figure 5:**
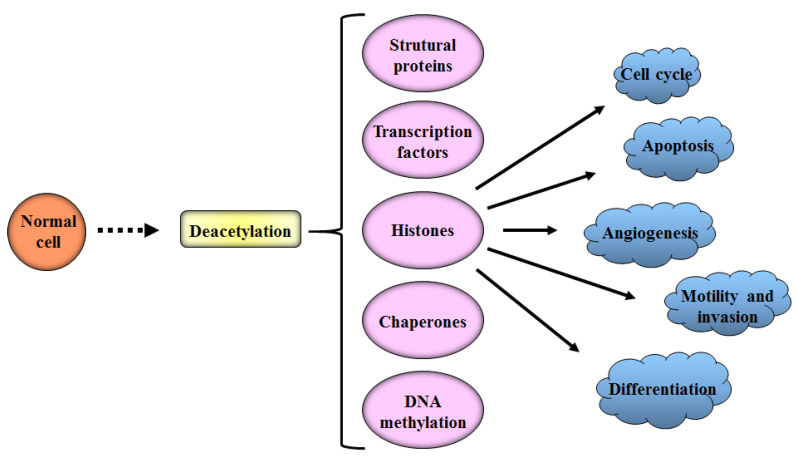
Some pathways that are altered by the activity of histone deacetylases. Acetylation and deacetylation of histones alter the chromatin activity, causing important epigenetic changes. In addition, the activity of non-histone proteins is altered, including transcription factors, chaperones and structural proteins, influencing the activity of the different pathways involved in the cell cycle control, apoptosis, differentiation, angiogenesis and cell invasion.

The effect of HDAC inhibitors (HDACis) is not restricted to histone proteins. These inhibitors can also target non-histone proteins, transcription factors, regulators, signal transduction mediators, DNA repair proteins and chaperones [183,184,185] (see Figure 5). There has been increasing interest in producing these drugs to better understand the functions of HDACs and to investigate the anticancer potential of these inhibitors [186].

Most HDACs have a Zn^2+^-dependent active site that can be inhibited by compounds with the ability to chelate this ion [187]. The currently used HDACis possess a pharmacophore that can bind to this active site and block it [188]. The preclinical efficacy of HDACis is associated with the gene activation mediated by these drugs, which promote the hyperacetylation of the N-terminal tails of histones, facilitating the access of transcription factors to gene promoters [180]. HDAC inhibitors can be natural or synthetic compounds that differ in terms of their target specificity [189]. Many structurally diverse compounds can bind to HDACs and inhibit their enzymatic activity. These compounds are classified into two large classes, isoform-selective inhibitors and pan-inhibitors, which act against all class I HDACs [134,190].

These inhibitors are divided into five subgroups based on their chemical structure: short-chain fatty acids that include hydroxamic acids, benzamides, cyclic peptides, and sirtuin inhibitors [19,176,191]. The functions of immune system cells with excessive accumulation of acetylated histones may be altered and it is therefore of the utmost importance to carefully select HDACis for the treatment of diseases such as cancer [175]. The inhibitors of HDACs render cancer cells more sensitive to immunotherapy, increasing the expression of antigens present in the tumor and thus acting as immunomodulators [192]. The anticancer activity of HDACis comprises different molecular and physiological events such as the inhibition of the vascular endothelial growth factor (VEGF), endothelial nitric oxide synthase (eNOS) and TGFβ1 [193,194,195]. The apoptosis of tumor cells induced by HDACis is associated with their ability to selectively regulate proapoptotic pathways [196], which does not occur in normal cells [197]. There are a number of different classes of HDACis which are available to treat cancers. 

**Figure 6:**
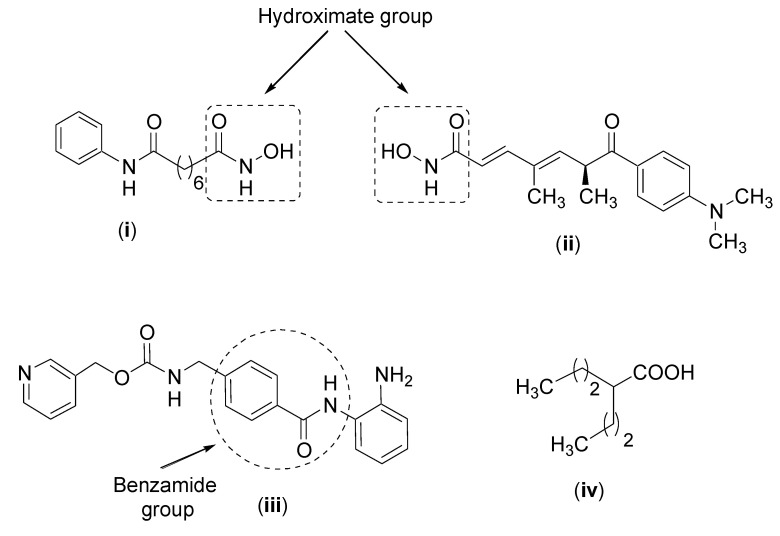
Structures of vorinostat (**i**), trichostatin A (**ii**), entinostat (**iii**) and valproic acid (**iv**).

First, there are the drugs which contain a hydroxamic acid group such as vorinostat [198], whose structure, and that of other HDACis, are presented in Figure 6. The function of the hydroxamic acid group is to chelate with zinc which is located in the active site of the enzymes. Another HDACi which contains the hydroxamic acid group is trichostatin A which is a highly toxic compound [199]. These compounds can inhibit all classes of HDACs. A second cluster of drugs which also interacts with zinc are benzamide derivatives, as exemplified by entinostat, which inhibits class I HDACs. A third group of HDACs are short chain fatty acids such as valproic acid which has been widely used in treating epilepsy. It inhibits classes I and IIa HDACs. In addition, butyric acid and phenylbutyric acid inhibit classes I and II HDACS [184]. Another HDACi is romidepsin, which is a cyclic tetrapeptide and a class I inhibitor [200]. 

HDACis have shown very promising results for the treatment of various neoplasms and several in vitro [201,202,203,204,205] and in vivo [206,207] studies have sought to understand the methods of action and the pathways involved in the anticancer process of these molecules. To date, only vorinostat (SAHA), romidepsin (FK228), panobinostat ((LBH589), belinostat (PXD101) and chidamide (CS055/HBI-8000) have been approved for clinical trials in the United States. These compounds show antineoplastic activity in the treatment of several hematological malignancies [208,209,210,211,212,213,214]. Class III-directed HDACi target NAD^+^-containing sirtuins and have shown an effect in the treatment of cardiovascular and neurodegenerative diseases and aging [215]. Variable biological effects of HDACis have been observed, which are the result of the individual chemical structure and profile of each inhibitor [199].

Although HDACis are showing important activities, mainly oncological, their adverse effects and cytotoxicity are still serious and do not present selective inhibition among HDACs isoforms [216]. To design an effective HDACi, the molecule must have synergy with other anticancer agents given that the HDACi used as a single agent does not show clinical benefits in nearly all types of solid tumors [217]. The involvement that HDACis have in the levels of epigenetic alteration for which they are responsible explains why they are so involved in altering normal phenotypes in malignant ones. Figure 6 contains the structures of four HDACis but others are currently under development.

## 7. Alternative Inhibitors

Despite their benefits which were proven in clinical trials, synthetic HDACis still have undesirable adverse effects [200]. One alternative is the search for natural products and their derivatives that are able to inhibit epigenetic changes caused by changes in gene expression, with less risk to the patient [218]. Psammaplin A is a natural product derived from bromotyrosine that is found in marine sponges [219]. This compound has been shown to inhibit the activity of HDACs and DNA methyltransferase, exhibiting low cytotoxicity in in vitro studies [220]. Largazole is a macrocyclic depsipeptide isolated from marine cyanobacteria, which exerts antiproliferative activity by inhibiting HDACs 1, 2, 3 and 6 [221,222]. This compound is a promising pro-drug for the treatment of carcinomas. Several natural products exhibit low HDAC inhibitory activity, but modifications in their chemical structure can produce analogs with high inhibitory activity. FK228 (FR901228), also known as a depsipeptide (a peptide in which one or more of its amide groups are substituted by an ester), is produced by *Chromobacterium violaceum* and exhibits HDAC inhibitory potential and antitumor activity in vivo [223]. Several natural compounds appear to interfere with most of the molecular mechanisms that involve cell proliferation and death. Natural compounds isolated from plants such as polyphenolic compounds, for example fisetin [224], resveratrol [225], curcumin [226] or flavonoids [227], can induce epigenetic changes, increasing the sensitivity of cancer cells to chemotherapeutic agents, and reduce tumor proliferation. Resveratrol is a natural, biologically active polyphenol that is found in grape seeds and peanut skin and that has therapeutic applications in the treatment of a range of diseases, including cancer [228,229,230]. Resveratrol can reverse the progression of prostate cancer by inhibiting MTA1 that binds to HDAC, forming the MTA1/HDAC complex [231]. This naturally occurring HDACi inhibited the activity of 11 HDACs in hepatoblastoma cells [232], the concentration-dependent histone hyperacetylation in hepatoma cell lines and cytotoxicity, but only at high doses [233]. Combination treatment with resveratrol and other HDACis revealed important antitumor activity in leukemia [234] and ovarian [235] and pancreatic carcinomas [174].

### 7.1. Chalcones

Chalcones (1,3-diaryl-2-propenones) are intermediates in the biosyntheses of flavonoids and isoflavonoids. They can be synthesized by the condensation between aryl aldehydes and acetophenone [236,237]. Their structure consists of two aromatic rings linked by a three carbon unsaturated keto group [236]. Chalcones are found in medicinal plants, fruits, vegetables, spices and nuts, and have anti-inflammatory [238], antihistaminic [239], antihypertensive [240], antidiabetic [241], antimalarial [242], antiretroviral [243], antioxidant [236] and antitumor [237] properties. In regards to the antitumor properties of chalcones, one should note that they are far less toxic than many current anticancer drugs [244]. In an in vitro and in vivo study, different chalcones synthesized in the laboratory were effective against colon adenocarcinoma, altering epigenetic pathways and inhibiting HDACs [245]. In computer-assisted studies on the activity of chalcones, β-hydroxymethyl chalcone exhibited the best time-dependency (∼24 h) as a broad-spectrum HDACi and β-hydroxymethyl chalcone as a selective inhibitor of HDAC2 [246]. Evaluating the inhibitory activity of 21 natural chalcones, researchers found the significant HDAC inhibitory activity of four, namely isoliquiritigenin, butein, homobutein and marein, against class I, II, and IV HDACs [247]. The 3,2,3’,4-tetrahydroxychalcone inhibited the class III HDAC SIRT1, resulting in tumor suppression [248]. Chalcones exhibited significant anti-proliferative activity against the HDAC inhibitory activity in carcinoma cell lines when compared to the synthetic drug SAHA, which is already used clinically [249]. These data suggest that natural compounds are promising in cancer treatment.

### 7.2. Curcuminoids 

**Figure 7:**
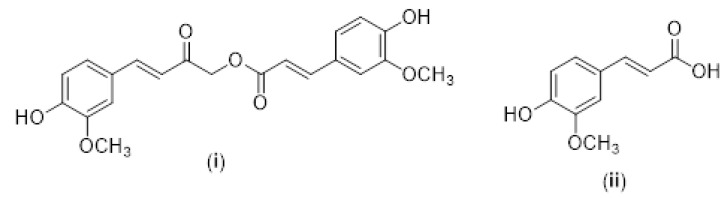
Structures of calebin A (**i**) and ferulic acid (**ii**).

Another potential HDACi is curcumin (diferuloylmethane), a polyphenol and the active component of turmeric (*Curcuma longa*) [250], which is widely known for its diverse pharmacological activities against various diseases, including cancer [251,252,253,254,255]. Turmeric is composed of 80% curcumin, 17% dimethoxycurcumin and 3% bisdemethoxycurcumin [256]. Curcumin can alter several important molecular signaling pathways that are responsible for cell survival and inflammatory responses and for reducing the expression of genes such as the tumor necrosis factor, adhesion molecules, interleukins (IL-1, IL-6, IL-8), C-X-C chemokine receptor type 4 (CXCR-4), and C-reactive protein [257,258,259]. Curcumin is considered a DNA hypomethylating agent that inhibits DNA methyltransferase and balances the activity of HATs and HDACs (HDAC 1, 3, 4, 5, 8) [260]. This compound was first described as a specific inhibitor of the coactivator p300/CBP. The latter interacts with numerous transcription factors and has been shown to increase the activity of acetyltransferase in cervical cancer cells. Its inhibition induced apoptosis [261]. Several HDACis have been used for the treatment of cancer alone or in combination with chemotherapeutic agents. Curcuminoids have shown deacetylase inhibitory activity that can suppress DNA repair pathways and can be used to increase the efficacy of cancer treatments [262]. A reduction of HDAC1 and HDAC3 activity was found in lymphoblastic cells treated with curcumin [263]. Furthermore, curcumin significantly reduced class I HDAC levels and increased acetylation [264,265]. Calebin A-([(*E*)-4-(4-hydroxy-3-methoxyphenyl)-2-oxobut-3-enyl]-(*E*)-3-(4-hydroxy-3-methoxyphenyl)prop-2-enoate) is a curcumin analog that contains a ferulic acid ester bond [266] (see Figure 7). 

This compound can be used as an adjuvant in cancer treatment, increasing the efficacy of the chemotherapeutic agents used; however, its bioavailability is low [267]. The inhibition of HDACs promotes cell death and inhibits angiogenesis in different tumor cell lines in in vitro studies [268] (see Table 2). 

Curcumin increases the sensitivity to DNA damage, reduces the repair of double-strand breaks and inhibits homologous recombination by inhibiting HDACs and promoting the degradation of recombinases [262]. In another study, in addition to interacting with the active zinc-containing site, curcumin exhibited an excellent inhibition of class 1 and 3 HDACs [275]. The enzymatic activity of HAT was found to be reduced in tumor cells treated with calebin A [276]. However, another study demonstrated that calebin A inhibited HDAC1, similarly to curcumin [277]. Tetrahydrocurcumin is a curcuminoid obtained by the reduction of curcumin. It is synthesized in the laboratory by hydrogenation but can be produced in vivo by metabolism in the liver [278]. Tetrahydrocurcumin did not inhibit HDACs [277], probably because its mechanism of action differed from that of calebin A and curcumin which have similar mechanisms. However, this curcuminoid exhibits anti-inflammatory potential and antioxidant activity which may explain its anticancer activity [279]. Chalcones have been linked to HDACs in an attempt to better understand the mechanisms of action of these deacetylases, with epigenetic importance in the treatment of diseases [247]. A molecular docking study concluded that curcumin does not bind to HDAC8 through the interaction with the zinc ion; this deacetylase is inhibited by the interaction with Arg37, Pro35, Ile34 and Phe152 residues located in the active site of the enzyme [280].

## 8. Conclusions

This review has outlined the structures and functions of different classes of histone deacetylases (HDACs). A major emphasis has been placed on various inhibitors of HDACs and how they exert their bioactivity. Although neoplasms are currently the main clinical indication for these compounds, future applications may include autoimmune diseases, neurological indications and even parasitic diseases. However, improvements in the therapeutic index of these drugs should be made, as they present high toxicity, inducing symptoms from fatigue, nausea and vomiting to thrombocytopenia, neutropenia and some cardiac toxicity. One path to this improvement may come from more specific inhibitors of individual HDAC isoforms that are critically involved in particular indications. By targeting the most relevant HDAC isoform in a specific indication, it may be possible to significantly improve their efficacy by removing certain toxicities that may be associated with the inhibition of multiple isoforms [281].

## Figures and Tables

**Table 1:** Type, class, protein size and localization of histone deacetylases (HDACs).

HDAC Type	HDAC Class	Amino Acids	Cellular Localization	Cancer Type	Referencers
HDAC1	I	482	Nucleus	Lung, gastric, liver, breast, ovarian, prostate, renal, bladder, hematological tumors	[52,53,54,55,56,57,58,59]
HDAC2	I	488	Nucleus	Medulloblastoma, gastric, pancreatic, colorectal, breast, ovarian, prostate, renal, bladder, hematological tumors	[54,55,56,57,58,60,61,62,63]
HDAC3	I	428	Nucleus and cytoplasm	Lung, gastric, breast, ovarian, prostate, bladder, melanoma, Hematological tumors	[54,55,57,58,62,64,65]
HDAC4	IIa	1084	Nucleus and cytoplasm	Hematological tumors	[58]
HDAC5	IIa	1122	Nucleus and cytoplasm	Hematological tumors	[58]
HDAC6	IIb	1215	cytoplasm	Hematological tumors	[58]
HDAC7	IIa	912	Nucleus and cytoplasm	Hematological tumors	[58]
HDAC8	I	377	Nucleus, Mitochondria and cytoplasm	Neuroblastoma, melanoma, hematological tumors	[58,65,66]
HDAC9	IIa	1011	Nucleus and cytoplasm	Hematological tumors	[58]
HDAC10	IIb	669	Cytoplasm	Cervical, chronic lymphocytic leukemia	[59,67]
HDAC11	IV	347	Nucleus	Lung	[68]

**Table 2:** In vitro studies on the effects of curcumin on histone deacetylases (HDACs).

Cell Line	Curcumin	Molecular Effects	Result	References
Lymphoma Raji cells	1.6–50 μM	Cell proliferation	↓HDAC1 mRNA and protein expression	[269]
Human cervical cancer cell lines	0.5–50 μM	Augments the efficacy of antitumor drugs	↓HDAC activity, ↓HDAC1and 2 expression	[270]
HepG2 cell line	100 μM of different curcumin analogues	Modulates genes	↓HDAC1, 2, 4, 6, 8, 11 expression	[271]
K562, HEL, and MPN cell lines	5–40 μM	Increases the expression of suppressor of cytokine signaling 1 and 3	↓HDAC activity ↓HDAC1, 3, 8 expression	[265]
LNCaP cells	5 μM	CpG demethylation	↓HDAC activity ↑HDAC1, 4, 5 and 8 and ↓HDAC3 protein expression	[272]
HT29 cell line	2.5 and 5 μM	Inhibitory effect on cell proliferation	↓HDAC4, 5, 6 and 8 expression	[273]

Adapted from [274].
